# Visualizing the drivers of an effective health workforce: a detailed, interactive logic model

**DOI:** 10.1186/s12960-021-00570-7

**Published:** 2021-03-12

**Authors:** Serena Sonderegger, Sara Bennett, Veena Sriram, Ummekulsoom Lalani, Shreya Hariyani, Timothy Roberton

**Affiliations:** 1 Johns Hopkins Bloomberg School of Public Health, 615 N. Wolfe Street, Baltimore, MD 21205 USA; 2 University of British Columbia, Vancouver, BC Canada

**Keywords:** Health policy, Human resources for health, Health workforce, Health services administration and management, Governance, Evidence-to-policy, Framework, Logic model

## Abstract

**Background:**

A strong health workforce is a key building block of a well-functioning health system. To achieve health systems goals, policymakers need information on what works to improve and sustain health workforce performance. Most frameworks on health workforce planning and policymaking are high-level and conceptual, and do not provide a structure for synthesizing the growing body of empirical literature on the effectiveness of strategies to strengthen human resources for health (HRH). Our aim is to create a detailed, interactive logic model to map HRH evidence and inform policy development and decision-making.

**Methods:**

We reviewed existing conceptual frameworks and models on health workforce planning and policymaking. We included frameworks that were: (1) visual, (2) comprehensive (not concentrated on specific outcomes or strategies), and (3) designed to support decision-making. We compared and synthesized the frameworks to develop a detailed logic model and interactive evidence visualization tool.

**Results:**

Ten frameworks met our inclusion criteria. The resulting logic model, available at hrhvisualizer.org, allows for visualization of high-level linkages as well as a detailed understanding of the factors that affect health workforce outcomes. HRH data and governance systems interact with the context to affect how human resource policies are formulated and implemented. These policies affect HRH processes and strategies that influence health workforce outcomes and contribute to the overarching health systems goals of *clinical quality*, *responsiveness*, *efficiency*, and *coverage*. Unlike existing conceptual frameworks, this logic model has been operationalized in a highly visual, interactive platform that can be used to map the research informing policies and illuminating their underlying mechanisms.

**Conclusions:**

The interactive logic model presented in this paper will allow for comprehensive mapping of literature around effective strategies to strengthen HRH. It can aid researchers in communicating with policymakers about the evidence behind policy questions, thus supporting the translation of evidence to policy.

## Background

In health systems research, practice, and policy over the past two decades, various goals have consistently been articulated—including improved health, responsiveness, efficiency, and social and financial risk protection [1]. As one of the health systems “building blocks”, the health workforce plays an indispensable role in achieving these goals [2–4]. Globally, there is increased focus on expanding the availability, accessibility, acceptability, coverage, and quality of the health workforce as a critical step to achieving universal health coverage (UHC) and the Sustainable Development Goals [2]. Strengthening the evidence base for workforce policies and the uptake of evidence-informed policies are critical to achieving these goals [2].

In recent years, researchers have made strides in examining issues such as recruitment strategies, training, supervision, and outcomes such as retention, motivation, and distribution [5–7]. To make this growing literature more accessible to policymakers, frameworks can help consolidate research to demonstrate factors affecting workforce outcomes, strength of linkages, and interrelationships that impact the health workforce as part of a robust health system. In turn, such a visual consolidation of evidence can provide researchers feedback on areas with existing evidence and those requiring greater attention. Ultimately, this could create a two-way interaction between researchers and policymakers facilitating the evidence-to-policy translation process in the context of human resources for health (HRH) policymaking.

There are multiple frameworks that explore the determinants of health workforce performance [8–13]. Most include some elements of contextual factors, health system building blocks, planning and implementation, and processes across the HRH lifecycle leading to HRH goals—but portray these elements and their relationships in different ways. The majority of frameworks are developed for decision-makers (e.g., government officials or leaders), showing how they can influence HRH outcomes to achieve health system goals—for example, through influencing health labor markets [9, 14] or using HRH “action fields” to advance policy objectives [8].

While these frameworks provide useful guidance on HRH policies and policymaking, they are generally high-level and conceptual. They show broad causal pathways, but most do not show causal relationships between *specific* factors, or the strength of the relationships between these factors. We have growing evidence on these specific relationships, sometimes including measurement of effects [7, 15, 16]. Other HRH frameworks focus on specific workforce outcomes or cadres of health workers [17, 18] without overarching interactions across domains.

In this paper, we present a detailed logic model that synthesizes existing HRH conceptual frameworks to (1) depict how upstream context, governance, and policy decisions affect the HRH lifecycle and downstream HRH outcomes and impacts, and (2) provide a base for mapping the evidence for these interactions, allowing for exploration of research and policy pathways. To bring the logic model to life, we use an interactive visualization platform that allows the logic model to be expanded and collapsed to show different levels of detail. Although such platforms are still new, they have many potential applications; for example, the Lives Saved Tool (LiST) Visualizer allows users to explore the relationships captured by the LiST modeling tool—which uses mathematical modeling to estimate the impact of changes in intervention coverage on mortality in low- and middle-income countries (LMICs) [19]. While in the past, a comprehensive yet detailed model showing specific relationships and their strength would not have been feasible given technological and space constraints, an interactive online “visualizer” tool addresses these barriers. An interactive tool also allows for overlaying other data on the model; for example, allowing users to click on elements of the model to explore the available evidence and appropriate indicators.

We see potential for this type of interactive tool to expand the traditional concept of a framework or logic model, creating possibilities for otherwise static images to become portals to explore data [20, 21]. In the context of HRH, this tool could support the evidence-to-policy translation process, synthesizing research in a visually appealing and accessible way for policymakers and practitioners. It could also help bridge the gap between academia, policy, and practice, while advancing global health workforce goals [2].

This paper first outlines the methods used for developing the logic model and visualizer tool. Next, we describe the conceptual frameworks analyzed in developing the logic model and present the resulting high-level and detailed logic model. Finally, we discuss the utility of the visualizer in consolidating evidence and answering policy questions.

## Methods

We used a multi-stage process that included: (1) searching for existing HRH frameworks and selecting those that fit inclusion criteria; (2) reviewing the resulting frameworks and synthesizing their contents into a detailed logic model; and (3) adapting this multi-level logic model into an interactive platform for visualizing relationships and evidence.

### Search for existing frameworks

We conducted a targeted literature search to identify relevant conceptual frameworks. Initially, the research team identified existing frameworks from their prior knowledge and experience. Seven conceptual frameworks were identified and used as a starting point for our analysis, as well as to develop search terms for further exploration.

We searched PubMed, Google Scholar, and Google Image, using combinations of “human resources for health” or “health workforce development” or “health labor market”, and “framework” or “conceptual model” or “conceptual framework” or “theoretical framework”. In addition, we reviewed websites consolidating guidance on HRH, including the Capacity Project resource page [22], WHO Health Workforce resource page [23], and CapacityPlus HRH Global Resource Center [24].

We used the following inclusion and exclusion criteria.

Inclusion criteria:

- Comprehensive frameworks aimed at supporting holistic decision-making around HRH policy, markets, and systems.
- Oriented towards improving health systems functioning and population health.
- Specific to the health sector.
- Includes a visual model (graphic conceptual framework).

Exclusion criteria:

- Narrowly focused frameworks targeting specific policy concerns (e.g. rural retention, balancing skill-mix of cadres, or external migration).
- Stepwise tools, guidelines, workforce projection equations, or similar, targeting specific components of HRH planning or policies.
- Frameworks focusing on a specific region or country that cannot be easily translated to other contexts.
- Close adaptations of frameworks already included in our analysis (to minimize overlap).

No date restrictions were applied.

### Review and synthesis

Information on the selected frameworks was collated and analyzed in an Excel matrix. Thematic categories were developed based on broad similarities across the frameworks: context and determinants; policy or action levers; health and HRH strategy planning and governance; processes across the HRH lifecycle; and ultimate goals and outcomes of HRH inputs and processes. We then mapped the different factors and subfactors for each conceptual framework into these thematic categories and sub-sections.

Analyzing the overlap and variations within the matrix, we synthesized categories, components, and relationships depicted in the conceptual frameworks into an initial comprehensive logic model. The high-level logic model visually depicts broad “hierarchical levels”, showing how upstream contextual factors, governance, and policy decisions affect processes across the HRH lifecycle and downstream HRH outcomes and impacts. The detailed logic model shows subcomponents and considerations within each. In addition to the frameworks identified in our search, we drew upon supplementary empirical research and literature to detail each component of our model (e.g., governance, political factors, etc.).

We also validated the initial model with two academic experts experienced in HRH policy-development and practice. These experts provided feedback on the layout, components, and potential future uses for the model.

### Interactive visualizer

We developed an interactive version of the model with the following features:

- An online software application, centered around a visual graphic of the logic model, built using scalable vector graphics (SVG) common to many web pages.
- Visualization of the logic model components (as “text boxes”) with nested subcomponents (“boxes within boxes”), with the ability to expand and collapse components.
- Visualization of relationships and interactions between components and subcomponents across different levels (i.e., upstream vs. downstream) using arrows.
- Ability to isolate impact pathways and relationships within the model, by focusing in on specific desired outcomes, interventions, or components and subcomponents.
- Ability to overlay information (e.g., research publications, indicators) onto components and subcomponents, so that users can click to reveal embedded references to empirical literature.

## Results

Our search and review process resulted in 10 conceptual frameworks on HRH that fit the inclusion and exclusion criteria. In this section, we (1) briefly outline these 10 frameworks and how they were incorporated into the resulting logic model, and (2) detail the resulting logic model developed based on these conceptual frameworks.

### Search results

**Fig. 1:**
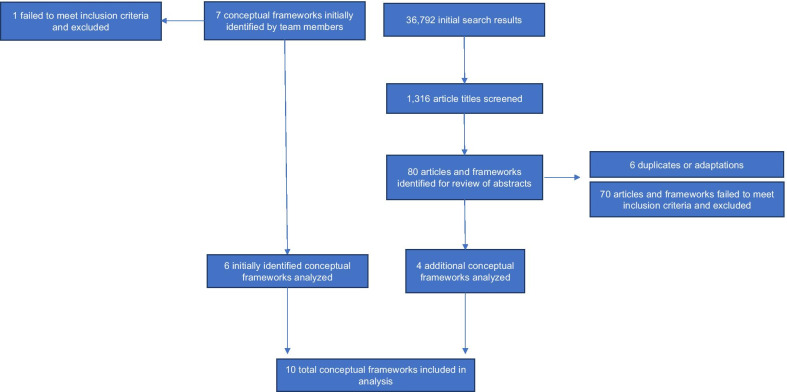
Selection strategy

The research team initially identified seven conceptual frameworks from the team’s existing knowledge. Six of these were included in the final analysis, while one did not meet inclusion and exclusion criteria. Our subsequent broader search yielded 36,792 results. Of these, the Google Scholar results yielded the highest number (between 160 and 20,400 per search). To manage the number of publications in the initial extraction, we reviewed the first ~ 150 titles for each search (total of 1316 articles across searches). Publications with titles that appeared to fit the inclusion and exclusion criteria were selected for further review (80 publications). We reviewed these 80 publication abstracts and found that 76 publications did not meet inclusion and exclusion criteria. Where frameworks were adaptations of other frameworks, we selected the most comprehensive framework for inclusion, or included both if they were sufficiently different in terms of components and focus. For example, [10, 25] are considered the same framework, while [8, 12, 26] are sufficiently different to warrant inclusion of all three. Four additional frameworks were identified, leading to a total of 10 frameworks summarized in Table 1. Figure 1 describes the selection strategy.

**Table 1:** Summary of frameworks

Name	Author	Year	Summary/objectives	Outcomes	Relation to the final logic model
HRH action framework [8]	World Health Organization (WHO) and Management Sciences for Health (MSH)	2008	Tool to guide policymakers and health managers to diagnose challenges with the health workforce (e.g., issues with shortages, distribution, competency, retention, and motivation) and determine solutions and implementation strategies to address underlying barriers, with the ultimate goal of improving workforce effectiveness and sustainability	Improved equity, effectiveness, efficiency, and accessibility of health services, leading to better health outcomes	“Action Fields” adapted to elements of the “Health system factors”
HRH in Fragile States [12]	Fujita et al	2013	Adaptation of the HRH Action Framework tailored for post-conflict, fragile health systems. Builds on HRH Action Framework by including more specific HRH policy and intervention areas to affect HRH outcomes in fragile settings	Human resource systems that are responsive to health needs	Foundational components of health system adapted to “Health system factors” HRH policy areas adapted to “Health workforce processes”
Developing the health workforce for universal health coverage [26]	Cometto et al	2020	Framework on individual, organizational, and systemic capacity-building for successful stewardship of HRH—building on HRH Action and systemic capacity-building frameworks [8, 64]. Shows health workforce policy levers at the individual and organizational levels and systems and contextual factors that are required for and enable effective HRH governance. Synthesizes effective policies for health workforce development within each HRH Action Field	Optimizing health workforce management to achieve UHC	Systemic capacity-building framework adapted to “HRH system governance”
Health worker productivity and performance [10]	Dieleman et al Also adapted by Global Health Workforce Alliance [25]	2006 2014	Logic model to depict strategies for improving the performance and productivity of the health workforce. Shows the interrelated mechanisms and contextual determinants that lead to health workforce outcomes, effects, and impacts	Improved performance and productivity (responsiveness, availability, and competency), leading to health improvements	Macro-level context adapted to “Contextual factors” Inputs adapted to “Health system factors” Processes adapted to “Health workforce processes” Outputs and Outcomes adapted to “Health workforce outcomes” Effects adapted to “Health system outcomes”
Systematic Approach to Health Workforce Management [28]	Dubois and Singh	2009	Framework for HR optimization, using a systems perspective to enhance the organizational unit. Management strategies should be aligned with one another and situated within the organizational environment and wider political, social, legal context—with individuals responding to organizational context, and organizations responding to policy environments	Organizational, staff, and patient outcomes	Institutional context adapted to “Contextual factors” Organizational context adapted to “Health system factors” Human resource management strategies adapted to “Health workforce processes”
Comprehensive health labor market framework for universal health coverage [26]	Sousa et al	2013	Stresses that policymakers should take a more comprehensive market-based approach, beyond simply training more health workers. Shows how policy areas can affect and interact with health labor market dynamics and the education sector—which in turn shape the distribution, pay, quality, performance, etc., of the health workforce	Workforce able to deliver quality health services to achieve UHC	Education sector, labor market dynamics, and societal drivers adapted to “Contextual factors” Policy areas adapted to “Health workforce processes”
Framework with health workers at the core of the health system [11]	Anand and Bärnighausen	2011	Framework that depicts health workers as the central element of a functioning health system, arguing that all functions of the health system depends on health workers, their activities, and the system elements that influence them	Appropriate size, composition, and distribution of health workforce providing access to treatment to improve population health and patient satisfaction	Health/HRH system inputs and mechanisms adapted to “Health workforce processes” Health workforce outcomes and activities adapted to “Health workforce outcomes”
Stages of Health Workforce Development [13]	WHO	2006	"Working Lifespan" approach for analyzing and responding to dynamics of the health workforce. Strategies and policy interventions relate to stages of health workforce entry, participation, and exit	Availability, competence, responsiveness, and productivity of the health workforce	Entry, Workforce, and Exit cycle and policy options adapted to “Health workforce processes” Workforce performance outcomes adapted to “Health workforce outcomes”
Imbalances in the health workforce [27]	Zurn et al	2004	Depicts the factors that affect imbalances in the health workforce (supply and demand of health labor, the health care system, policies, resources, and "global" factors). Develops policy tools to address these factors based on a typology of imbalances: profession/specialty imbalances, geographical imbalances, institutional and services imbalances, and gender imbalances	Correct health workforce imbalances	Wider context adapted to “Contextual factors” Policies adapted to “HRH policy formulation and implementation” Health Care System and Resources adapted to “Health system factors”
Framework for analysis of health workers labor market dynamics [14]	McPake et al	2013	Uses health labor market analysis (predominantly supply and demand analysis) to unpack factors behind HRH constraints, to more effectively design policies that can affect health labor markets and subsequent employment conditions. Focused on accelerating progress towards UHC	Improved quality of care and productivity of health workforce, increasing overall health systems performance	HRH regulation and governance adapted to “Health system factors” HRH management, motivation, incentives, and training, education, migration, retirement, deaths adapted to “Health workforce processes” Supply for HRH and HRH employment adapted to “Health workforce outcomes” HRH performance adapted to “Health system outcomes”

The 10 selected frameworks have similar high-level elements, but differ in level of detail, layout, and depicted relationships. Overlapping elements include contextual factors, health systems building blocks, HRH policy planning and implementation, processes that influence outcomes across the HRH lifecycle, and intermediate and ultimate HRH outcomes.

The frameworks differ in perspective, focus, and the outcomes they describe. Some see the labor market and/or individuals within the labor market as drivers of health workforce outcomes, and show how policies can be implemented to influence individual choices, the labor market, and/or the education sector to achieve desired health workforce size and quality [9, 11, 13, 14, 27]. Others look at more “top-down” approaches regarding HRH financing, planning, and policymaking [8, 12, 26]. Finally, some focus on organizational and contextual factors and management strategies that improve performance and outcomes [10, 28].

In addition to the frameworks, we identified several publications that did not meet our criteria (particularly that of a visual framework), but nonetheless provided insights about HRH governance and policies that informed our logic model. They are referenced in the logic model explanation that follows.

### Logic model

We adapted the common elements and relationships depicted in the frameworks into a detailed, interactive logic model, available at hrhvisualizer.org [29]. The final model has five columns with components (17), subcomponents (44), and interactions between them, which allows for high-level and detailed exploration of the model elements. The arrows in the model are directed and causality flows from left to right. We intentionally chose to create the model as a directed acyclic graph (DAG) and to minimize feedback loops. Although other models emphasize causal loops [30, 31], our goal was to show how upstream factors affect downstream outcomes. Although in reality these relationships are complex, our approach makes it easier to identify the “drivers” of an effective HRH workforce. Without this DAG constraint, the complexity of the model might overwhelm its clarifying purpose.

**Fig. 2:**
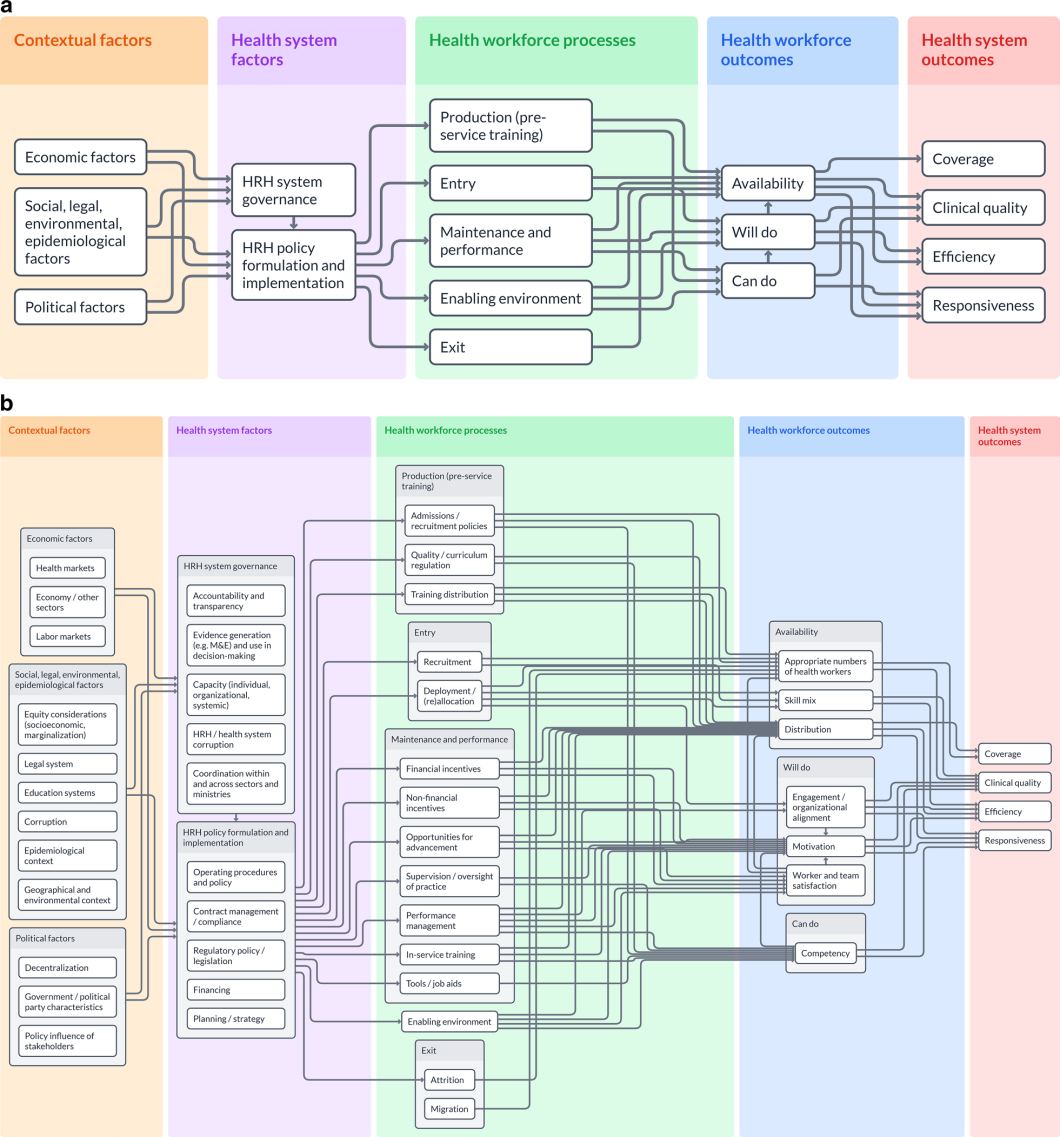
**a** High-level logic model. **b** Detailed logic model

The high-level logic model, shown in Fig. 2a, includes only the top-level components, and the broad linkages across columns. Each of the top-level components has additional subcomponents, which can be seen in Fig. 2b. The detailed model dives deeper and allows the user to see granular relationships between components and subcomponents.

Each of the columns, components, and subcomponents are described below.

Column 1. Contextual factors: these include factors within the broader societal, economic, political, and policy environment that affect downstream decisions and outcomes within the health and HRH system [32]. We categorized contextual factors into three larger components, following adaptation of the Political, Economic, Social, Technical, Legal, and Environmental (PESTLE) analysis framework.

- Social, legal, environmental, and epidemiological factors: the legal system (regulation and law enforcement); the basic and professional educational system; epidemiological and environmental factors that affect population distribution and health needs; levels of equity and/or marginalization of different groups (e.g., socioeconomic, gender, race, ethnicity, education, etc.), and levels of corruption [33].
- Economic factors: strength of the economy, nature of health markets—including levels of care, financing, and service provision—and larger labor market forces that affect health workforce distribution, public and private decision-making, and HRH outcomes.
- Political factors: decentralization of power and decision-making; the type and capacity of political regime in power and its level of “political entrenchment”; and the influence governmental and non-governmental stakeholders have on policy adoption and implementation [34].

Column 2. Health system factors: effective governance institutions, organizations, practices, and capacities for management, decision-making, and data use in policymaking support sound policy development and implementation. Specific areas of health and HRH systems and processes include:

- HRH system governance: leadership, processes, and capacities for governing HRH systems. This includes: individual, organizational, and systemic capacity for management and decision-making; collaboration and coordination within and across sectors and ministries for multisectoral action; transparency and accountability to government and communities; and corruption within the HRH and broader health systems [35–41]. Electronic human resource management systems (e.g., managing postings, performance, attendance) along with systemic capabilities to use data for workforce planning, regulation, and decision-making are important aspects of governance [36, 37]. Governance is affected by a wider national and international context, and influences HRH policy formulation and implementation.
- HRH policy formulation and implementation: areas of strategy and planning around health workforce objectives; financing allocations and mechanisms to implement HRH interventions and policies; regulatory policy and legislation around medical practice and health workers (e.g., scope of practice for each cadre, and/or expansion of functions); contract compliance of entities engaged to provide health services; and day-to-day operating procedures. Policymaking and implementation are affected by factors of HRH governance.

Column 3. Health workforce processes: HRH policies affect and are implemented through HRH processes.^1^ HRH processes and strategies may be implemented across four stages:

- Production of health workers: policies or factors influencing pre-service training of health workers which are generally used to affect the size, composition, competencies, and distribution of HRH. These could include admissions policies or regulation of the quality and distribution of training institutions—whether public or private [43].
- Entry into the health workforce: recruitment strategies and equitable distribution (deployment, reallocation) of the workforce per health system needs and across public and private sectors. Foreign-trained workers may also enter into the health workforce in some countries, dependent on regulations. The stock of health workers trained and the supply of workers currently functioning in a labor market may be different (due to exit from the health labor market, or inability to find work in the health labor market).
- Maintenance and performance: strategies used to retain health workers (particularly in remote underserved area) and manage their performance. These include clinical quality interventions (e.g., job aids or tools to support quality and work flow), performance management systems to measure and develop health worker performance [44, 45], in-service training to maintain and strengthen competencies, supportive supervision, and regulation of practice to ensure quality of care. In addition, it includes financial and non-financial incentives to promote health worker motivation, performance, and retention in remote underserved areas [46–51].
- Enabling environment: HRH outcomes will be affected by the environment within which the workforce operates. This includes healthcare facilities and infrastructure, availability of supplies and equipment, other health systems building blocks, and living conditions (i.e., road infrastructure, housing, and quality of local schools) [43, 48, 49, 52].
- Health worker exit: exit from the workforce can be a natural progression (e.g., retirement), attrition due to pursuit of alternative careers, migration to locations with better living and working conditions, or a lack of sufficient incentives or motivation to remain in the health workforce.

Column 4. Health workforce outcomes: workforce outcomes—specifically, health workforce availability, competency, motivation, engagement, and job satisfaction—are influenced by upstream policies, processes, and contextual factors [32, 46–52].

- Availability: availability of health workers to ensure geographic coverage according to population health requires having appropriate numbers of health workers, equitable distribution across urban and rural areas, and skill-mix across the cadres of healthcare workers.
- Will do: worker and team satisfaction, engagement, and motivation are critical for the competencies of health workers to translate into practice. “Engagement” refers to vigor and energy devoted to one’s work; involvement, dedication to, and enthusiasm in work; and absorption and identification with one’s work [53, 54]. Motivation and engagement of health workers support a drive towards quality and improving health outcomes, which supports responsiveness, efficiency, and equity of care [1, 55]. This is known as the “know–do” gap between provider skills and their application of these skills when delivering services [56]. These factors are also important for influencing retention of health workers.
- Can do: required competencies (knowledge, skills, and attitudes) that are critical for health workers to provide care with high clinical and non-clinical quality, based on designated roles and responsibilities.

Column 5. Health system outcomes: desired health system outcomes focus on “improving health and health equity, in ways that are responsive, financially fair, and make efficient use of available resources” [1, p. 2]. The ultimate goals of the health workforce are to contribute to these overall health system goals, by enhancing quality, responsiveness, efficiency, and coverage. If health worker performance is combined with a well-functioning health system, the workforce can deliver high-quality health services equitably, leading to improved population health. Specific health system outcomes the workforce contributes to include:

- *Quality* of service delivery in accordance with predefined standards and protocols, including clinical quality and non-clinical aspects such as safety and equity [2].
- *Responsiveness* in how the health system meets expectations around provider treatment [57]. This may encompass aspects of non-clinical quality including people-centeredness and patient satisfaction [2].
- *Coverage* of health workers across both urban and rural areas according to population health needs.
- *Efficiency* in utilizing financial and non-financial inputs, including appropriate skill-mix based on available human resources.

## Discussion

In this paper, we present a detailed, interactive logic model to inform HRH policy-development and research agendas. The visualizer is online for external input, and we anticipate subsequent revisions as we move forward in gathering further feedback and synthesizing evidence.

The model synthesizes existing frameworks and literature into a user-friendly interface that enables both high-level and detailed examination of policy areas. We see it complementing existing tools identified in this review [8–14, 26–28] by providing granular analyses of relationships and synthesizing underlying literature into a comprehensive model. In our discussion, we consider uses of the current model, describe potential future uses based on additional development, and identify specific use cases, before reviewing limitations.

### Current uses

In its current form, the model enables exploration of the factors driving HRH outcomes and the contribution of the workforce to health system outcomes.

- Evidence-to-policy process: we see the visualizer being useful in supporting the evidence-to-policy translation process because it provides a visually engaging and comprehensible format for exploring evidence behind key policy questions. Several barriers have been identified in the translation of research for policymaking, including succinct communication of complex methods and ideas, and insufficient time and capabilities for unpacking academic papers and understanding their implications on local context [58–60]. This tool can help address this gap and make the evidence behind policy options more comprehensible for policymakers.
- Education and training: by synthesizing current HRH frameworks and their linkages, this tool can illustrate higher-level policy pathways and relationships between HRH system components. It will allow learners to explore the pathways by which upstream factors and external forces affect the HRH lifecycle, and by which HRH processes contribute to health system outcomes.

### Future uses

We plan to overlay the model with evidence (e.g., research publications, systematic reviews) related to the model’s components, subcomponents, and relationships (arrows). Users will be able to click to reveal embedded references to empirical literature. For example, a list of publications could be added to the arrow between financial incentives and distribution of health workers, so a user can understand the empirical evidence behind this relationship.

The model also allows for visual depiction of evidence strength by using boxes and arrows of different weights, sizes, and colors. For example, a relationship that is hypothesized but not yet shown empirically might be represented by a dashed arrow, whereas a linkage that has been demonstrated in numerous studies might be characterized with a thick arrow. This feature could help to inform policy questions and research agendas.

We see multiple opportunities for the visualizer to add value once overlaid with evidence:

- Synthesizing and building health workforce evidence: the visualizer can be used to build consensus around existing and missing evidence on strengthening the health workforce, thereby serving as a dynamic platform that bridges the gap between evidence, policy, and practice. Researchers can use this tool to receive feedback from policymakers for directing future research questions, to ensure they can be of value to policymakers.
- Developing mathematical models: increasingly in global health, stakeholders want to quantify the potential impact of alternative policy options, or the resources required to achieve targets. Mathematical modeling has an important contribution to make in this regard, but until now, most HRH modeling has involved associative models that do not describe the causal pathways between factors [61–63]. Future modeling could involve causal models, in which mathematical models are grounded in an a priori understanding of how upstream determinants interact to affect a workforce and achieve health outcomes. This tool can serve as a base for developing such mathematical models.

### Illustrative use cases

In this section, we show two illustrative use cases of the visualizer. The first shows isolation of a “policy pathway” through which contextual factors and policy interventions affect rural health worker attraction and retention. The second describes an evidence map behind rural attraction and retention based on one publication [43].

**Fig. 3:**
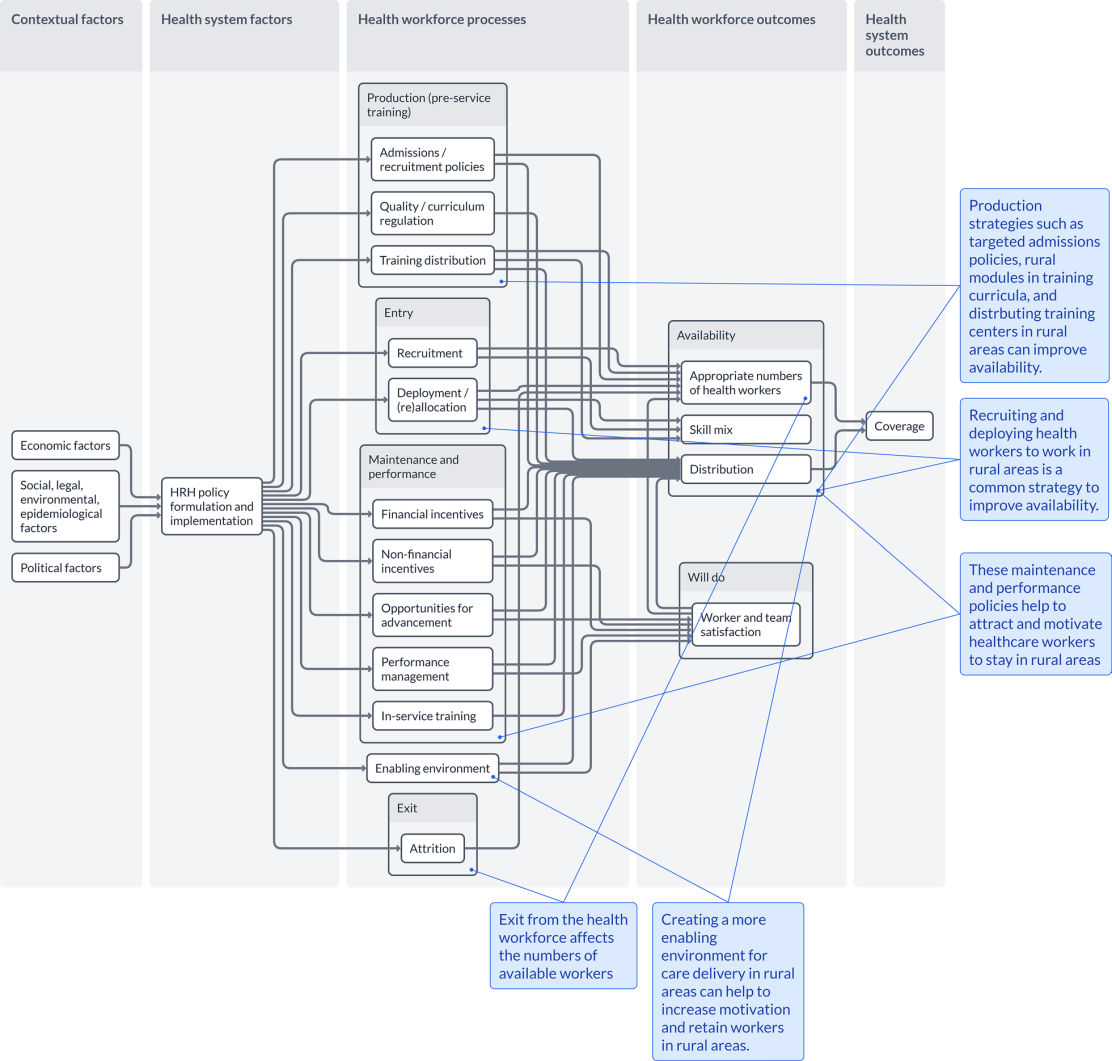
Example policy pathway—rural attraction and retention

Figure 3 depicts processes and strategies across the HRH lifecycle that affect rural distribution, as well as contextual factors that affect policy implementation. Callout boxes help to explain relationships, and related publications are shown when a user clicks on a specific factor or arrow.

**Fig. 4:**
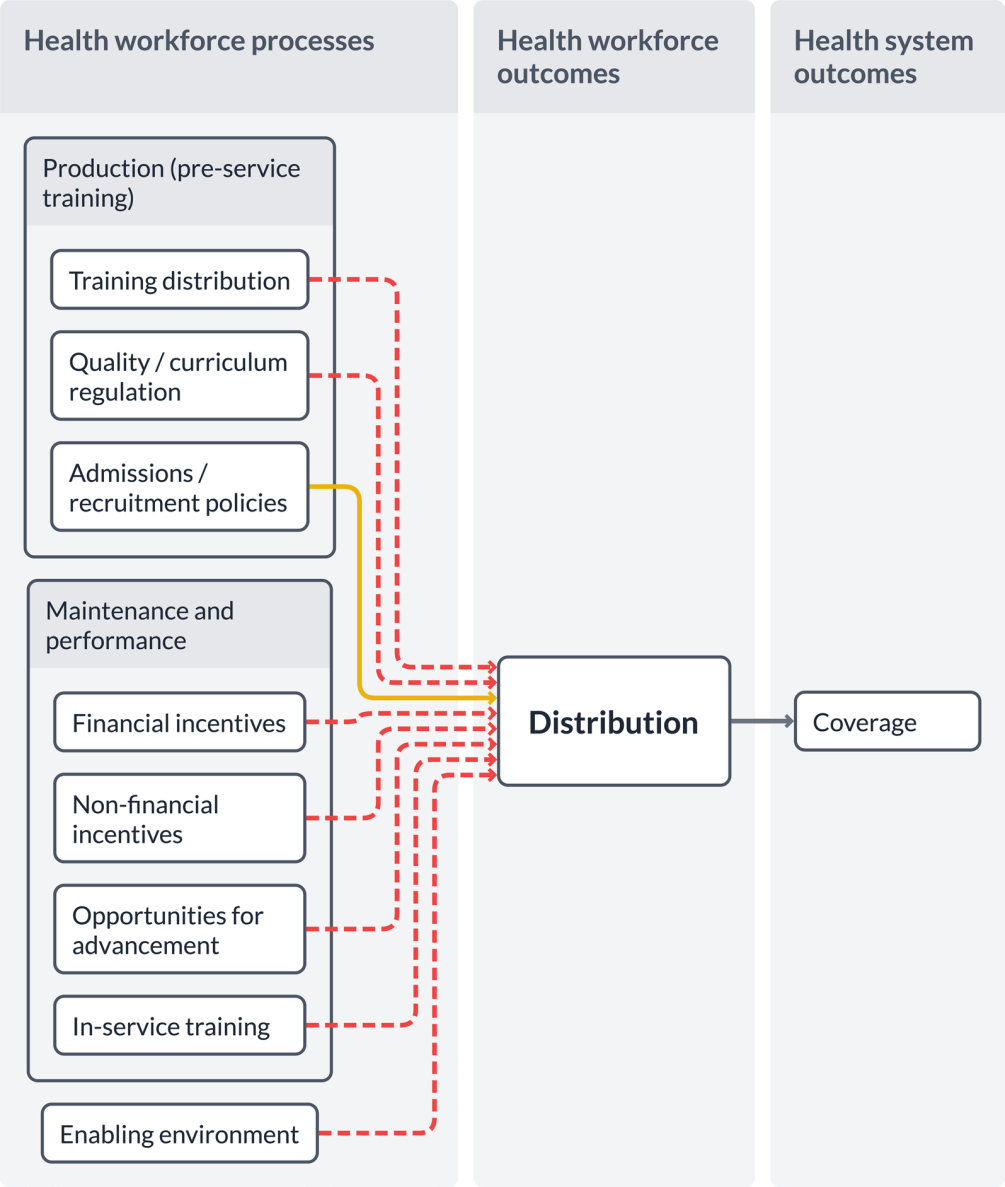
Example illustration of strength of evidence—rural attraction and retention

Figure 4 shows how the visualizer can be used to map evidence. We have laid out the evidence portrayed in the WHO’s policy recommendations for attracting and retaining healthcare workers in rural areas [43]. The dotted red line means “low” or “very low” quality of evidence, while a solid orange line means “moderate” quality of evidence.

These examples show the multiple potential uses for the visualizer platform, both now and in the future as evidence is mapped onto the model.

### Limitations

This analysis and the visualizer have limitations. Our review focused on a subset of databases and English-language publications, and therefore might have omitted relevant frameworks. Further, we recognize it is challenging to capture system complexity within a two-dimensional model. While a logic model requires components to flow from inputs to outcomes in a linear manner, reality is more complicated, with systems interactions and causal loops between upstream and downstream factors, and adjustments over time. For example, the model does not show context and governance’s direct effects on the other areas of the model—but rather show their impacts as mediated through policy formulation and implementation. Bringing in these feedback loops would add significant complexity to the model, but may detract from its ability to cleanly depict evidence.

## Conclusion

We built on existing research and conceptual frameworks to create a detailed, interactive logic model that shows the drivers of health workforce performance, and how an effective health workforce contributes to health system goals. The model allows users to see the policy levers and contextual factors that affect HRH processes and outcomes. We hope the tool will help researchers map out the concepts and literature within their area of study, and better understand the available evidence and research gaps. Our goal is to develop a collaborative tool for policymakers, academics, and practitioners, to help illuminate the field of HRH research, and build a shared understanding of steps to achieve health for all.

## Data Availability

Logic model visualizer available online [29].

## Abbreviations

DAG: Directed acyclic graph
HRH: Human resource for health
LiST: Lives Saved Tool
LMIC: Low- and middle-income countries
SVG: Scalable vector graphics
UHC: Universal health coverage
WHO: World Health Organization

## Footnotes

1 The Health Worker Life Cycle model [42] is another framework with similar “stages” of the lifecycle, which is based on Sousa et al. [9] and therefore was not included in our results.
